# Supplementary material for: Multidimensional analysis of immune responses identified biomarkers of recent Mycobacterium tuberculosis infection
Source: PLoS Comput Biol. 2021 Jul 28;17(7):e1009197. doi: 10.1371/journal.pcbi.1009197 (PMC8351927; doi:10.1371/journal.pcbi.1009197)
Supplement: S1 Table — (PDF) [file pcbi.1009197.s001.pdf]

Variable		Persistent QFT+ n = 30	Recent QFT+ n = 29	p-value [1]
Age in years at enrolment (median, range)		15 (13-18)	16 (13-18)	0.489
Female sex (n, %)		21 (70%)	18 (62%)	0.713
Ethnicity (n, %)	Mixed ancestry (coloured)	26 (87%)	28 (97%)	
	Black	4 (13%)	1 (3%)	0.371
School (n, %)	D	15 (50%)	18 (62%)	
	E	11 (37%)	9 (31%)	
	F	4 (13%)	2 (7%)	0.570
BMI (median, range)		20.72 (14.70-28.67)	19.34 (13.29-32.26)	0.394
Known TB exposure (n, %)	Ever	7 (23%)	9 (31%)	
	< 1 year of enrolment	4 (13%)	2 (7%)	0.710

[1] For age and BMI, Wilcoxon's non-parametric test was used to compare the median values between the persistent and recent QFT+ groups. For the remaining variables, a chi-squared test was used as a test of association.
