# Supplementary material for: Multidimensional analysis of immune responses identified biomarkers of recent Mycobacterium tuberculosis infection
Source: PLoS Comput Biol. 2021 Jul 28;17(7):e1009197. doi: 10.1371/journal.pcbi.1009197 (PMC8351927; doi:10.1371/journal.pcbi.1009197)
Supplement: S2 Table — (PDF) [file pcbi.1009197.s002.pdf]

Method	Explanation	Author(s)	R package
missForest	A non-parametric imputation method that uses Random Forest models to predict the missing values and can handle mixed data	Stekhoven et al. (2012) [1]	missForest [2]
k nearest neighbours (KNN)	An imputation method that employs the KNN algorithm to group variables with similar profiles. A weighted average of the K nearest complete variables is taken and used to impute the missing values in the incomplete variables	Troyanskaya et al. (2001) [3]	DMwR [4]
Column median	Missing values were simply imputed with the column median of the incomplete variable		
Multiple Factor Analysis (MFA)	Builds an MFA model on the incomplete dataset and uses the model to predict the missing values.	Husson et al. (2013) [5]	missMDA [6]

References

1.Stekhoven DJ, Buhlmann P. MissForest—non-parametric missing value imputation for mixed-type data. Bioinformatics. 2012;28(1):112–118.
2. Stekhoven DJ. missForest: Nonparametric Missing Value Imputation using Random Forest; 2013. R package version 1.4.
3. Troyanskaya O, Cantor M, Sherlock G, Brown P, Hastie T, et al. Missing value estimation methods for DNA microarrays. Bioinformatics. 2001;17(6):520–525.
4. Torgo L. DMwR: Functions and data for “Data Mining with R”; 2015. R package version 0.4.1.
5. Husson F, Josse J. Handling Missing Values in Multiple Factor Analysis. Quality and Preferences. 2013;30(2):77–85.
6. Husson F, Josse J. missMDA: Handling Missing Values with Multivariate Data Analysis; 2017. R package version 1.16.
