# Supplementary material for: Multidimensional analysis of immune responses identified biomarkers of recent Mycobacterium tuberculosis infection
Source: PLoS Comput Biol. 2021 Jul 28;17(7):e1009197. doi: 10.1371/journal.pcbi.1009197 (PMC8351927; doi:10.1371/journal.pcbi.1009197)
Supplement: S1 Text — Supporting information on PBMC isolation, stimulation and staining, and on the data pre-filtering methods. (PDF) [file pcbi.1009197.s003.pdf]

Supplementary Methods: Multidimensional analysis of immune responses identified biomarkers of recent *Mycobacterium tuberculosis* infection

Tessa Lloyd[1,2], Pia Steigler[1,3], Cheleka A.M. Mpande[1], Virginie Rozot[1], Boitumelo Mosito[1], Constance Schreuder[1], Timothy D. Reid[1], Mark Hatherill[1], Thomas J. Scriba[1], Francesca Little[2◑], Elisa Nemes[1◑*], and the ACS Study Team¶

1 South African Tuberculosis Vaccine Initiative, Institute of Infectious Disease and Molecular Medicine, Division of Immunology, Department of Pathology, University of Cape Town, Cape Town, South Africa
2 Department of Statistical Sciences, University of Cape Town, Cape Town, South Africa
3 Wellcome Centre for Infectious Diseases Research (CIDRI) in Africa, Institute of Infectious Disease and Molecular Medicine and Division of Immunology, Department of Medicine, University of Cape Town, Cape Town, South Africa

◑These authors contributed equally to this work.
¶Membership list can be found in the Acknowledgments section in the main text.
* elisa.nemes@uct.ac.za

1 Supplementary Methods

1.1 Data pre-filtering

1.1.1 COMPASS

COMPASS [1] was used to pre-filter the adaptive dataset by removing binary cytokine combinations that were not biologically meaningful. The filtering protocol was applied to CD4+ and CD8+ T cells for each antigen specificity separately. A subset was classified as biologically meaningful if the number of observations with posterior probability values greater than 0.1, at any one of either month 0, 6, 12 or 18, was greater than 10 (one third of the number of participants in one cohort). The posterior probabilities are used to quantify the likelihood of detection of Ag-specific responses. For example, E6C10-specific CD4+ T cells with a joint expression of all five cytokines would be omitted from the final dataset because the number of observations with posterior probabilities greater than 0.1, at all sampling occasions, is less than 10 (S1 Fig).

1.1.2 MIMOSA

We employed MIMOSA [2] to identify which individuals had a significant antigen-specific T cell response over background (unstimulated condition). Responding subjects are identified by testing whether the proportion of cytokine-producing cells in stimulated and unstimulated samples are different from each other. We defined as responders those individuals with a Th1 response in the stimulated samples that had a 3-fold change over unstimulated samples and a MIMOSA

false discovery rate p-value less than or equal to 0.01. Since background expression cannot be subtracted for phenotypic markers, we measured these markers only in stimulated samples from responder individuals.

1.1.3 Innate pre-filtering

COMPASS and MIMOSA have been designed to analyze antigen-specific T cell responses, with the assumption that background cytokine expression in unstimulated samples is generally very low. This is not the case for innate responses, where spontaneous cytokine production can occur even in absence of stimulation and could be biologically meaningful. To our knowledge, no similar computational tools exist that could handle variable and sometimes high background responses. In addition, we measured a variety of functional markers that could be expressed by multiple cell types, but we did not expect that all cell types would express all functional markers included in the panel (i.e. some measurable combinations are not biologically meaningful). Therefore, we established our own pre-filtering protocol for the innate dataset, summarized in S2 Fig.

We defined a threshold value that would identify whether a cell subset expressing different functional markers was detectable or not (i.e. biologically meaningful). Responses were considered as detectable if:

- the upper bound of the 95% confidence interval around the median, which was calculated across all samples, identified by bootstrapped methods, was non-zero, and
- one third of all samples have values greater than zero.

As a first step, this detection criterion was applied to the total cytokine variables. The goal was to identify which cytokines each cell type was able to produce in response to either *M.tb*-lysate or *E.coli* (the positive control for this dataset) stimulations. If the total cytokine variable was detectable in response to either *M.tb*-lysate or *E.coli*, then the variable would be retained. Otherwise, if the total cytokine variable was considered undetectable for both stimulations, then the variable was removed from the analysis of that cell type, including the binary functional combinations.

The second step focused on filtering the binary combinations of the different cytokines for each cell type. We first tested whether the binary subset was detectable when it was left unstimulated. If it was detectable, we further tested whether the *M.tb*-lysate stimulated version of this subset was significantly higher than when left unstimulated. If the lower bound of the 95% CI around the median for the *M.tb*-lysate sample was greater than the upper 95% CI of the median for the unstimulated version, the *M.tb*-lysate stimulated sample was kept in addition to the unstimulated sample. Otherwise, the *M.tb*-lysate stimulated sample was discharged and only values of the unstimulated version were kept. The rationale was that if the *M.tb*-lysate and unstimulated values were the same, then the biological responses were not different and hence it was unnecessary to keep both versions. If the unstimulated version of a cell subset was found to be undetectable, we

tested whether the *M.tb*-lysate stimulated version was considered detectable. If it was, then the *M.tb*-lysate version was kept, otherwise we discarded both versions.

Lastly, for *M.tb* lysate-specific subsets that were preserved post-filtering, we performed background subtraction by subtracting the values measured in the unstimulated sample from the same set (i.e. same individual and time point). The exception for this was when the cell subset was Granzyme B positive. As Granzyme B is a cytotoxic molecule that is constantly present in cells and not only expressed after stimulation, hence, it is not meaningful to perform background subtraction.

1.2 PBMC isolation, stimulation and staining protocol

PBMCs were isolated using CPT tubes (BD Bioscience), washed twice and cryopreserved in 50% RPMI, 40% fetal bovine serum and 10% DMSO in liquid nitrogen. Samples were collected between 2006 and 2008, and thawed between 2017-2019, therefore have been cryopreserved for 9-13 years. Average post-rest viability of thawed cells was excellent (97%).

To determine cytokine responses of innate, DURT and B cells (innate panel), cryopreserved PBMCs were thawed, washed and rested for 2 hours in R10 media [RPMI 1640 (Gibco), 10% fetal bovine serum (Gibco), 1% L-glutamine (Gibco) and 1% penicillin-streptomycin (Gibco)] prior to stimulation. Cells were then stimulated in R10 containing *M.tb*-lysate (H37Rv, 10 μg/mL, BEI Resources) to determine responses against mycobacterial antigens, heat killed *Escherichia coli* (*E. coli*, 10^7 bacilli per 1x10^6 cells, in house production) or left unstimulated (negative control). Cells were stimulated for a total for 6 hours at 37°C with 5% CO2. Brefeldin A (5μg/mL, Sigma Aldrich) and Monensin (2.5μg/mL, Sigma Aldrich) were added after the first 2 hours of stimulation and incubated for another 4 hours until harvest. After incubation, cells were detached from tubes using 2mM EDTA (Sigma Aldrich) in PBS (Lonza). Staining (Supplementary Table 1) for viability and surface markers was performed for 30 minutes at room temperature. Following surface staining, cells were washed, permeabilized and fixed (CytoFix/CytoPerm, BD Biosciences) for intra-cellular staining (ICS) of cytokines. ICS was performed for 30 minutes at room temperature. Prior to acquisition on a LSRII flow cytometer (BD Biosciences), cells were washed and fixed [1% paraformaldehyde (Kimix) PBS].

Supplementary Table 1: Antibodies for innate panel

Marker	Fluorochrome	clone	Company
CD3	BV786	UCHT1	BD
CD14	PerCpeF710	61D3	eBioscience
CD16	AF488	3G8	Biolegend
CD19	BV711	SJ25C1	BD
CD26	BV605	M-A261	BD
CD56	BV50	HCD56	Biolegend
GB	BV510	GB11	BD
CD161	PECy5	DX12	BD
$\gamma\delta$ TCR	BV421	11f2	BD
IL-10	PE-CF594	JES3-19F1	BD
IL-6	PE	MQ2-13A5	BD
IL-12	APC	C11.5	BD
IFN-γ	AF700	B27	BD
TNF	PECy7	MAb11	BD
Live Dead	Near-IR	-	LifeTechnologies

For the adaptive panel, PBMCs were processed and stained as described in [3].

References

1. Lin L, Finak G, Ushey K, Seshadri S, Hawn TR, Frahm N, et al. COMPASS identifies T-cell subsets correlated with clinical outcomes. Nature Biotechnology. 2015;33(6):610–616. doi:10.1038/nbt.3187.

2. Finak G, McDavid A, Chattopadhyay P, Dominguez M, De Rosa S, Roederer M, et al. Mixture models for single-cell assays with applications to vaccine studies. Biostatistics. 2014;15(1):87–101.

3. Mpande CAM, Rozot V, Mosito B, Musvosvi M, Dintwe OB, Bilek N, et al. Immune profiling of *Mycobacterium tuberculosis*-specific T cells in recent and remote infection. EBioMedicine. 2021;64:103233.
