# Supplementary figures and images for: Multidimensional analysis of immune responses identified biomarkers of recent Mycobacterium tuberculosis infection
Source: PLoS Comput Biol. 2021 Jul 28;17(7):e1009197. doi: 10.1371/journal.pcbi.1009197 (PMC8351927; doi:10.1371/journal.pcbi.1009197)

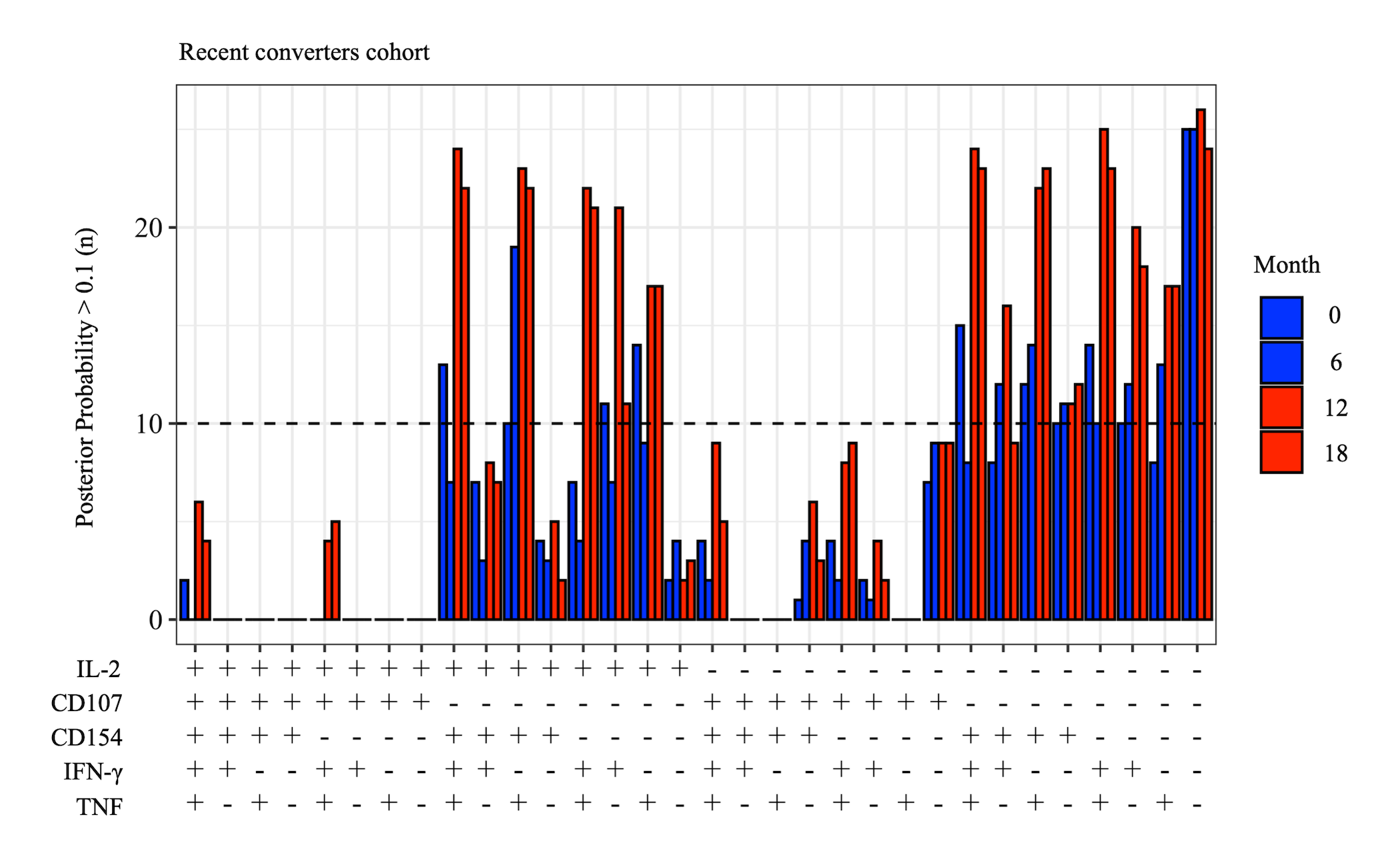

Supplement: S1 Fig — The number of observations for CD4+ T cell counts stimulated with E6C10 in the recent QFT+ individuals that had posterior probabilities (calculated by COMPASS) greater than 0.1 for each binary combination, stratified according to month. A subset was classified as biologically meaningful if the number of observations with posterior probability values greater than 0.1, at one of either month 0, 6, 12 or 18, was greater than 10 (one third of the number of participants in one cohort). (TIF) [file pcbi.1009197.s004.tif]

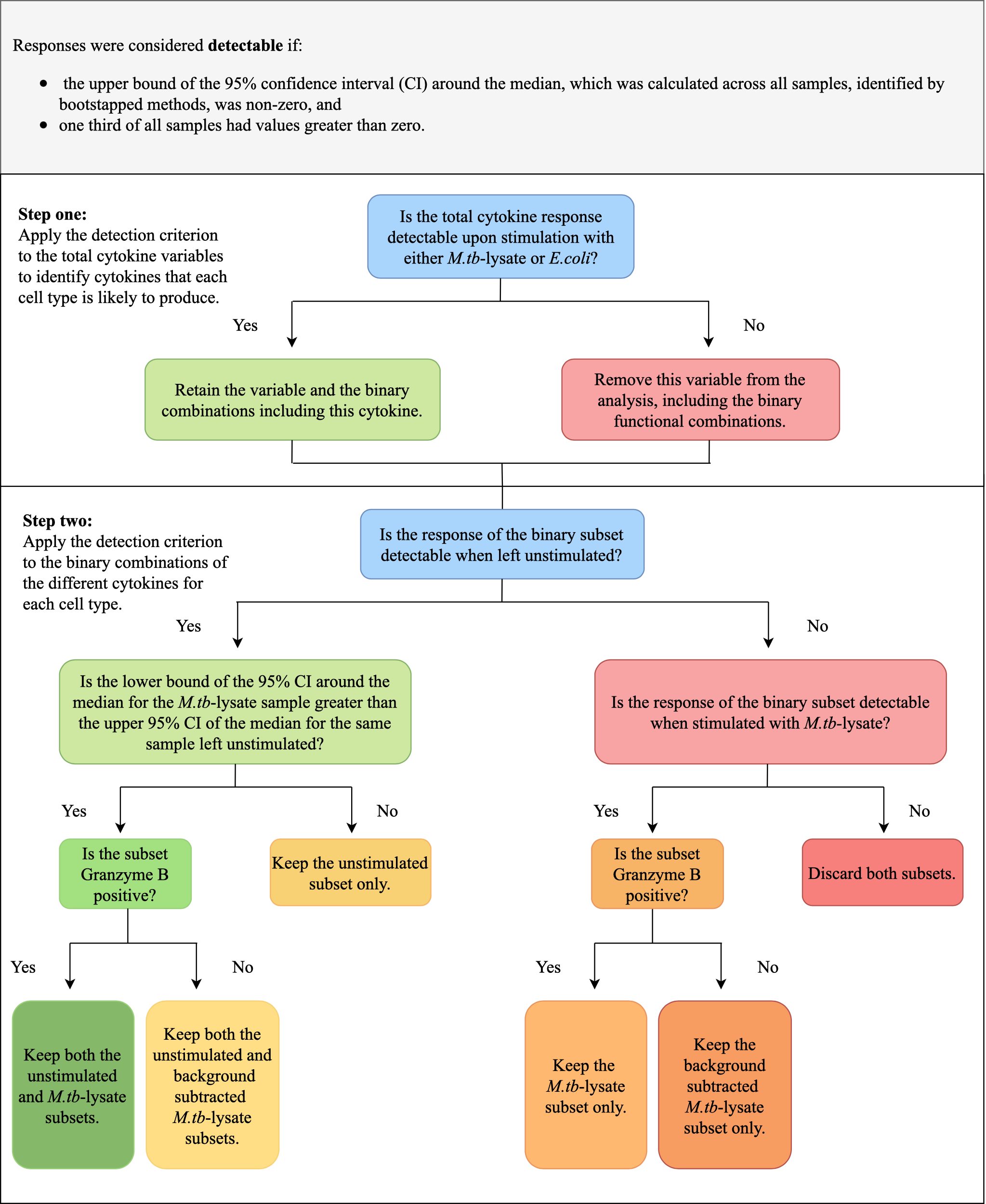

Supplement: S2 Fig — (TIF) [file pcbi.1009197.s005.tif]

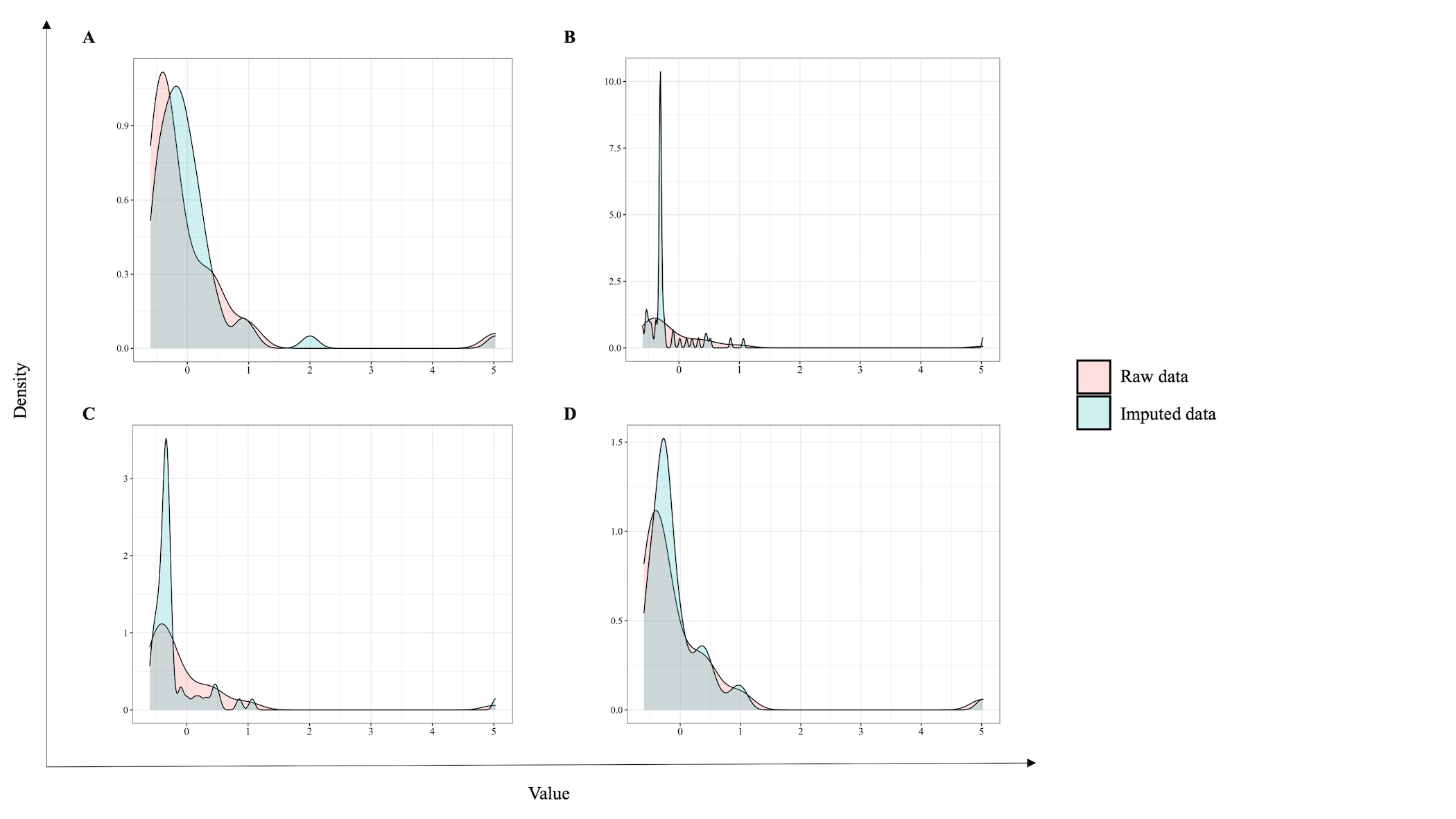

Supplement: S3 Fig — The efficacy of each imputation method to capture the distribution of the raw frequencies of total IFN-γ production in NKT cells stimulated with M.tb-lysate is shown as an example. The red lines are the raw data in each plot and the blue lines are (A) MFA, (B) column median, (C) k-nearest neighbours and (D) missForest imputed values. (TIF) [file pcbi.1009197.s006.tif]

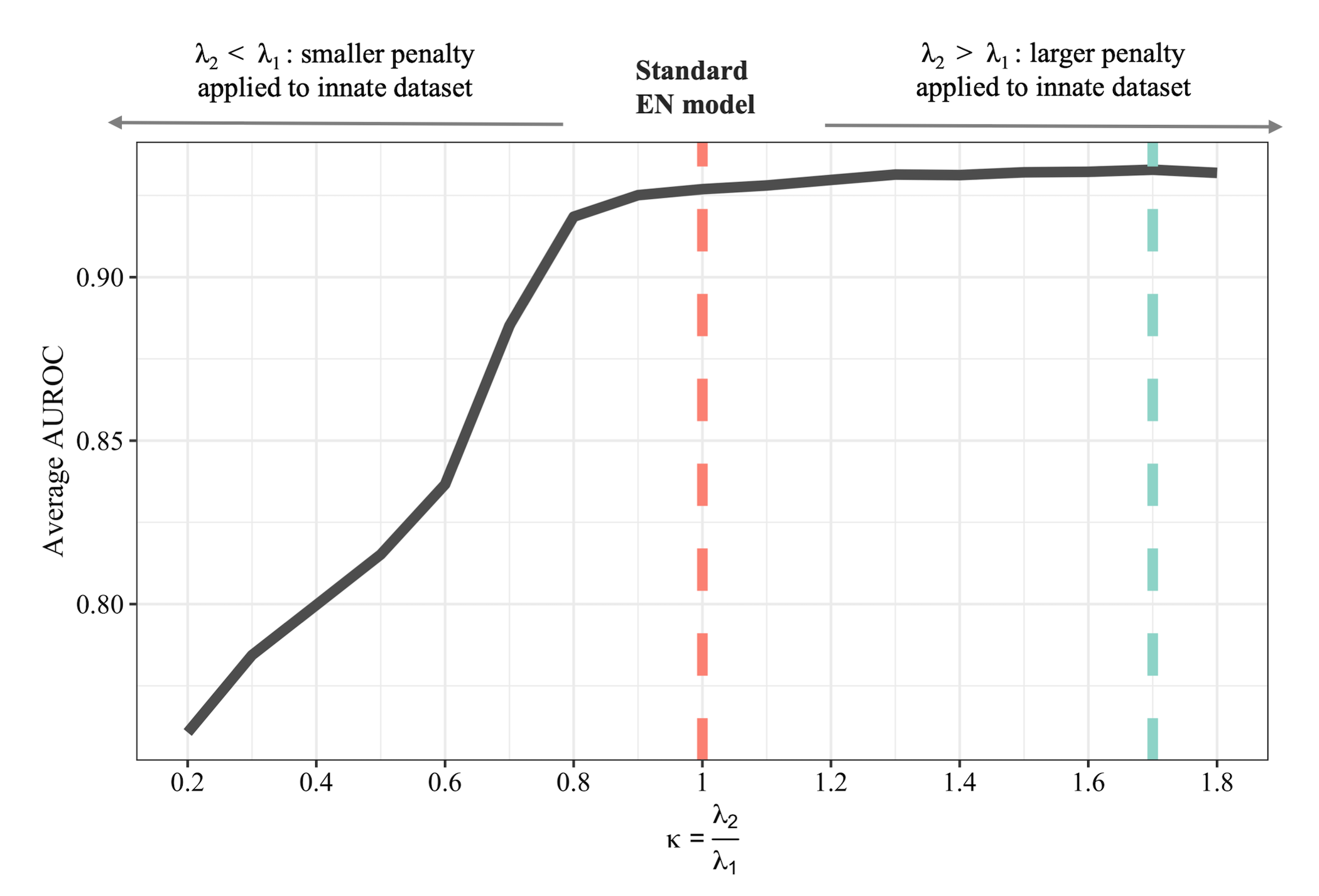

Supplement: S4 Fig — The average of 500 AUROC values is plotted as a function of κ, the ratio of the penalty parameter for the innate dataset relative to that for the adaptive dataset. When κ < 1 (λ2 < λ1) a smaller penalty is applied to the innate dataset, and when κ > 1 (λ2 > λ1) a larger penalty is applied to the innate dataset. A red dashed line is plotted at κ = 1 (λ2 = λ1), which is equivalent to a standard EN model, and a blue line at the “optimal” κ = 1.7, corresponding to the highest mean AUROC. (TIF) [file pcbi.1009197.s007.tif]

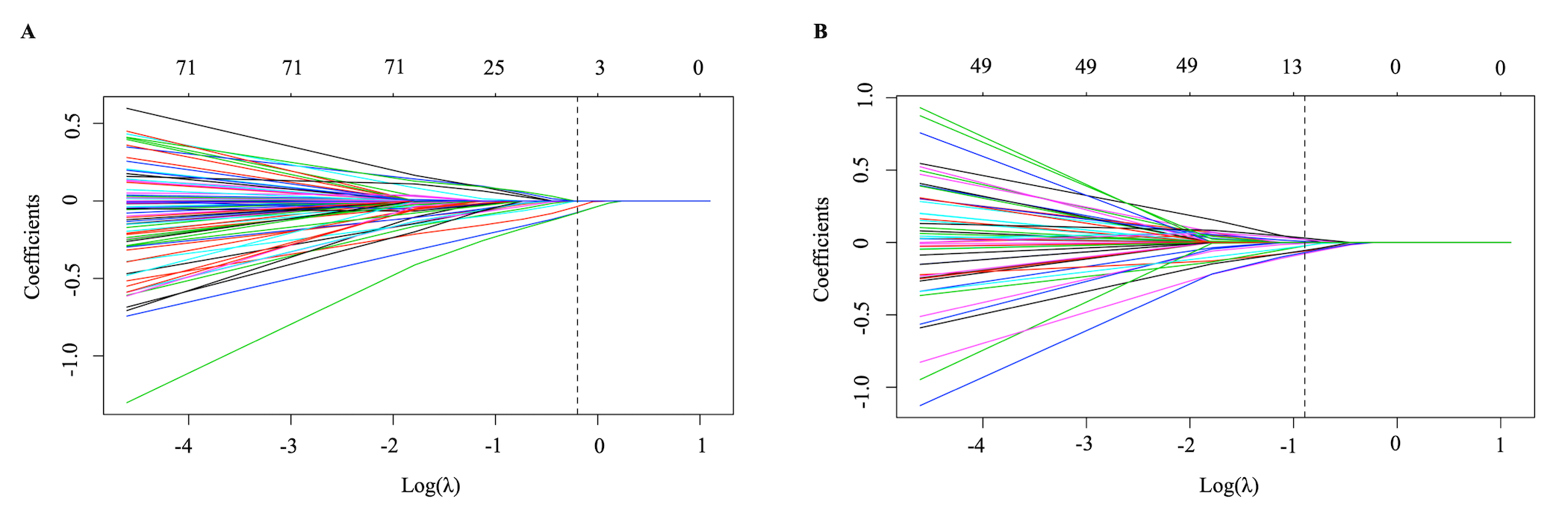

Supplement: S5 Fig — Each line in the plots represents the coefficients of one variable for different values of λ, the overall shrinkage parameter in the EN model, from the respective datasets. An increasing value of λ leads to the shrinkage of more regression coefficients and the number of non-zero coefficients for each λ value are shown at the top of the figure. In the adaptive EN model (A) α was set to 0.21, where α ≤ 1 is the weight given to the L1 penalty and (1- α) the weight to the L2 penalty. A dotted line is plotted at log(λ) = -0.2 (λ = 0.82), the optimal parameter values from the final adaptive EN model. At this point the number of non-zero coefficients are three and correspond to E6C10-specific or M.tb-lysate-specific HLA-DR expression on total Th1 cells and Esp-specific CD4+IL2+CD107-CD154-IFN-γ-TNF+ T cells. For the final innate EN (B) α was set to 0.21 and a dotted line is plotted at log(λ) = -0.89 (λ = 0.41) corresponding to 11 non-zero coefficients. (TIF) [file pcbi.1009197.s008.tif]

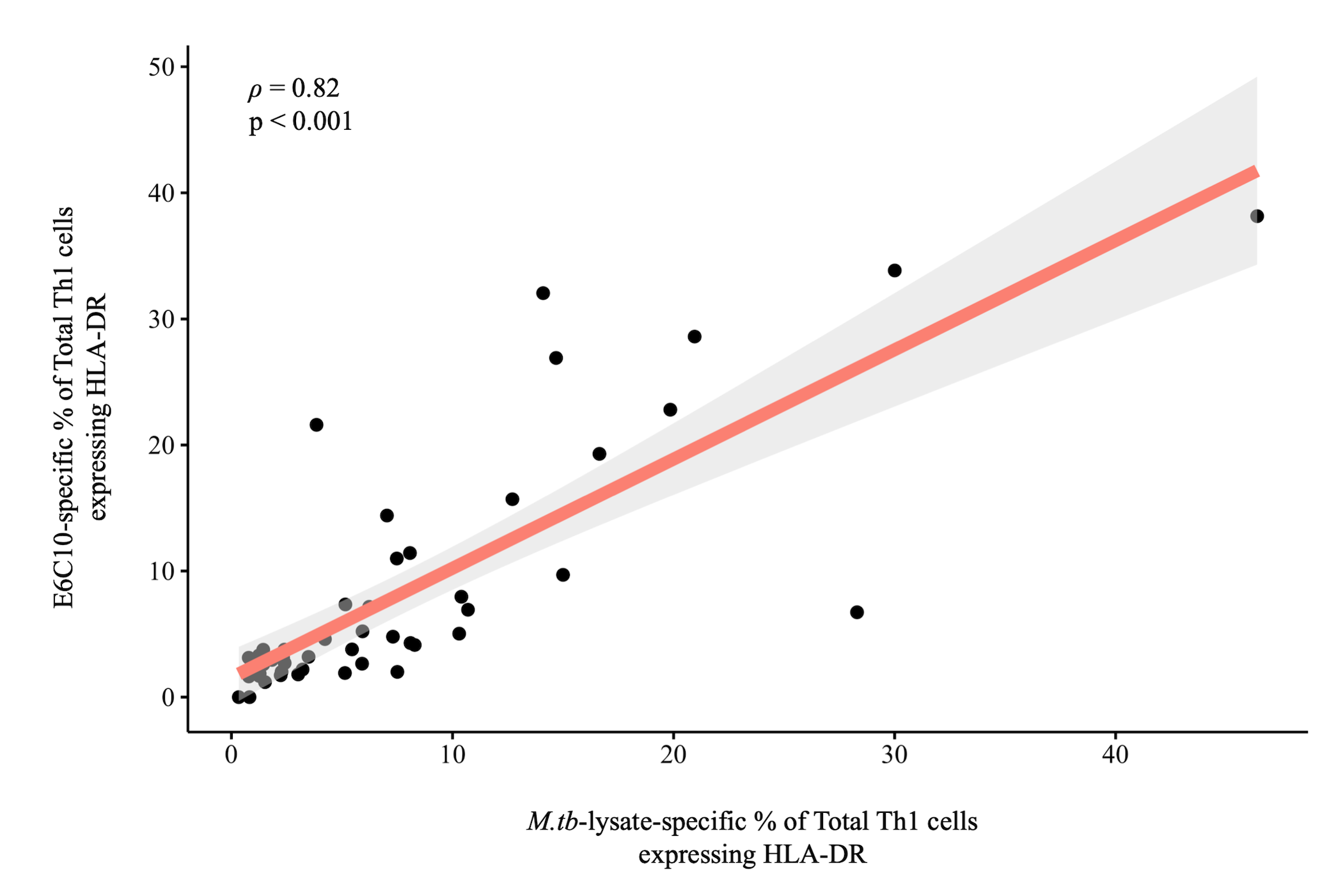

Supplement: S6 Fig — Spearman’s non-parametric correlation coefficient and its associated p-value are superimposed onto the plot. (TIF) [file pcbi.1009197.s009.tif]

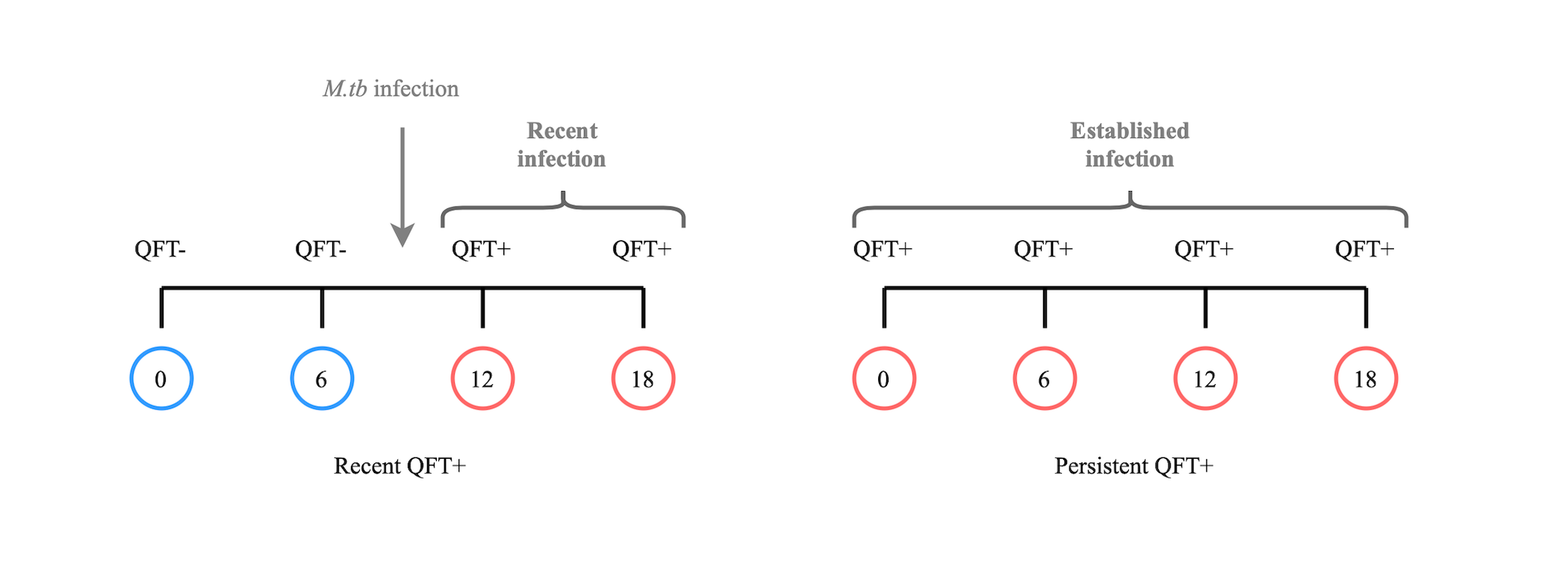

Supplement: S7 Fig — (TIF) [file pcbi.1009197.s010.tif]
