# Supplementary material for: Multidimensional analysis of immune responses identified biomarkers of recent Mycobacterium tuberculosis infection
Source: PLoS Comput Biol. 2021 Jul 28;17(7):e1009197. doi: 10.1371/journal.pcbi.1009197 (PMC8351927; doi:10.1371/journal.pcbi.1009197)
Supplement: Attachment — Submitted filename: Response to reviewers comments.docx [file pcbi.1009197.s011.docx]

Dear Editors,

We thank you for your and the reviewers’ thoughtful comments and the opportunity to address the concerns in our point-by-point response below.

***The results are interesting but a number of clarifications is needed. Please take into account all the reviewers comments. The cohort/patient population should be better described. It would be also beneficial for the paper to validate the results on an independent cohort. It also should be discussed which clinical parameters are used to correct the statistical analysis, and if no adjustments are done, explain why. It is also important to verify whether the model is not overfitted.***

RESPONSE: We have now included a comprehensive description of the cohort; results have been validated and published (reference 24, Mpande et al, Am J Respir Crit Care Med 2021 Jan 6. doi: 10.1164/rccm.202007-2686OC); we included discussion of clinical parameters and model overfitting. More specifically, for model overfitting we discuss how the performance of the final logistic regression model was validated via an internal validation procedure, and was found to be sufficiently high. In addition, the estimated coefficient HLA-DR was similar in the model with HLA-DR and Esp CD4+IL2+TNF+ cells and with HLA-DR as the only predictor, indicating that the relationship between this biomarker and recent Mtb infection is unchanged after variable selection. Lastly, we built the RF model to ensure that the results were generalizable across statistical methods.

**Reviewer #1:  This paper attempts to identify immune factors associated with recent TB infection, as opposed to long-term latent TB. The authors investigate TB-specific and non-specific responses in both innate and adaptive cell types in a cohort of longitudinally monitored South African adolescents. In particular, they point to an increase in HLA-DR+ T cells specific for ESAT-6 and CFP-10 and IL2+CD107-CD154-IFNg+TNF+ specific for EspC, EspF and Rv2348c as key markers of recent TB infection. These findings are both novel and important. However, the paper has a number of flaws, including a lack of clarity in the Results on the study cohort(s) and immune filtering criteria. The models described here were evaluated by cross validation, without an independent validation cohort. Without independent validation it is difficult to know how replicable these results would be in another cohort.

Comment 1: Line 124: Overall, the results section is lacking in important details. Line 124 states that “in order to successfully integrate the two datasets”. This is unclear. What are the datasets being referred to here? Innate and adaptive? One study dataset is described in the Methods, but a basic overview should be in the results. These include, numbers of participants, what samples were taken and what type of assays were run. The criteria for filtering the datasets (see also line 424 in methods – more detail needed) and selecting the imputation method should also be stated, these are key points for understanding the study and this information should not be relegated to supplemental methods.**

RESPONSE: We agree with the reviewer that the result section did not contain enough of the information explained in the methods, and we have added a brief description of the study groups (lines 112), clarified that data integration refers to the adaptive and innate datasets (lines 130 and 435), which were independently generated from samples collected from the same individuals, have added a brief rationale for the variable filtering (lines 133) and the reason for selecting the imputation method (lines 146).
 **Comment 2: Line 133: The MTP-EN model. The authors perform MTP-EN as well as standard elastic-net regression. MTP-EN provided no benefit over the elastic net, so I do not think this merits its own section. This could probably be relegated to a sentence in the subsequent section saying that MTP-EN does not provide any benefit.**

RESPONSE: This has been amended in the manuscript. We briefly introduce that an MTP-EN model was built to the data, as we believed the predictive performance of this model would be higher than the standard EN model. Since this was not the case, we agree that it does not merit its own section and have summarized accordingly.

**Comment 3: Line 154: The final EN models … identified three candidate biomarkers. What does ‘identified’ mean here precisely? Are these three markers the only non-zero coefficients in the model? See also Line 173, with 10 biomarkers**

RESPONSE: We used the word “identified” because the EN models were used as variable selection techniques in this setting, but we re-worded this section to indicate which variables had non-zero coefficients in the models.

There were only three non-zero coefficients in the EN model built to the adaptive dataset and the EN model built to the integrated dataset (both adaptive and innate variables). Both EN models yielded the same three non-zero coefficients.

**Comment 4: Line 159: The authors note that HLA-DR+ Th1 cells respond to either E6C10 or total Mtb lysate. What about the IL2+CD107-CD154-IFNg-TNF+CD4+ T cells? Do these also respond to TB lysate? Overall, do cell types that respond to specific TB antigens also respond to total lysate? This is an important control.**

RESPONSE: Yes, overall cell types that respond to specific TB antigens also respond to the total M.tb lysate, but frequencies of IL2+CD107-CD154-IFNg-TNF+CD4+ T responses to the lysate were not different between recent and remote infection.

**Comment 5: Line 230: Statistical Validation. The authors apply an alternate classification tree-based approach to the data and term this statistical validation. I do not understand how this validates their results. A true validation would require prediction on a separate set of data held out of the training process, and training another model on the same data to get a similar result is of little value. See also Line 348 in the discussion.**

RESPONSE: We agree that this is not a true validation and that was not our intent. Due to the various variable selection techniques that were applied to the dataset eg: building an EN model and using backwards variable selection, to get to the model that included HLA-DR and Esp-specific CD4+IL2+CD107-CD154-IFNg-TNF+ T cells, we used the tree-based methods as a method validation. We run the risk of overfitting the model using the variable selection techniques; therefore, we applied a different tree-based algorithm to ensure our approach was generalizable across statistical methods. We have re-worded accordingly (lines 351).

**Comment 6: Line 377: “Two cohorts of participants” Cohort normally refers to the entire group of participants in a study. The authors seem to mean “classes” rather than cohorts.**

RESPONSE: We agree with the reviewer and re-worded as two “groups” of participants, since all participants were from the same adolescent cohort, but were grouped and compared based on their QFT status (i.e. those who were persistent QFT+ versus those with QFT conversion).

**Comment 7: Line 385: The definition of recent QFT converters was limited to those who showed two negative QFTs followed by two positive QFTs. It is also possible that one negative followed by three consecutive positives or three negative followed by one QFT positive are recent converters. What would happen if these individuals were included in the study?**

RESPONSE: We agree with the reviewer that all these scenarios would also be considered recent infection. For this study, we applied a stringent definition of 2 consecutive negative tests followed by 2 consecutive positive tests to have a very robust definition of the clinical phenotype. The time from exposure to M.tb to conversion of QFT (or TST) is estimated to be ~40 days, which is the time required to develop a robust adaptive immune response that is measured by these assays. If we considered a single negative test before conversion, we could have accidentally included individuals who were already infected but had not yet converted their QFT test. Conversely, reversion of QFT results from positive to negative does occur (and still have unclear clinical significance), and therefore we included at least 2 consecutive positive tests to make sure that this was not the case during the follow-up of these participants.

**Comment 8: Line 418: “Integration was performed by aligning each dataset” But there is only one study cohort mentioned – what are the datasets that have to be aligned?**

RESPONSE: The adaptive and innate datasets were generated independently of one another and then aligned to allow integration of data generated by different assays on samples collected from the same individuals. This has been clarified (lines 435).

**Reviewer #2:  The manuscript provides a very detailed account of TB data analysis based on immuno markers of various kind. Their main results is the identification using ML techniques of one biomarker to discriminate between recent and persistent interferon measures.

Main comment: While the results are interesting and should be published, I find the manuscript very hard to follow overcrowded with details and key features missing. Below in random order the various comments/criticisms I have made for the manuscript:

Comment 1: The cohort is not well described and all the analyses are based on it. Even if great care has been taken to select the participant the study lacks details and statistical analyses to assure that other confounding factors does not bias the dataset.**

RESPONSE: We apologise for this omission. We cited another paper we recently published on the same cohort which contained detailed information. We have now added the same information in this manuscript (lines 112 and 396, and Supplementary Table 2).

**Comment 2: Related to previous comment: since all statistical analyses are performed based on the immune data only, no clinical parameters were introduced to correct the statistical analyses. This should be addressed either before hand or during the results comparisons.**

RESPONSE: We have now included this information (lines 112 and 396). By design, groups were balanced for the demographic variables that may have affected the QFT results (sex, age, ethnicity and school of recruitment and history of TB exposure) based on previous analyses we conducted on the larger cohort (n=6363) from which these participants were selected (reference 11, Mahomed et al., Int J Tuberc Lung Dis). Since all participants were healthy, we did not have other clinical variables to correct for in the statistical analysis. Further, no significant associations were found between these clinical parameters, which included BMI, and group status.

**Comment 3: It is not clear why the data integration is a challenge here since we seem to look at immuno data coming from the same experiments. There does not seem to have some batch effects nor clinical data. So i don’t understand.**

RESPONSE: The reviewer is right in saying that there are no batch effects. The main challenge was accounting for the vastly different scales of the two datasets (adaptive and innate immune responses, independently measured by different assays) and meaningfully imputing the missing rows that resulted because of the alignment step when not all variables were available. These are common problems that need to be addressed in data integration projects. Processing of data risks influencing and potentially biasing any results found in the final integrated model. Therefore, we emphasize the importance of testing different approaches and taking the time to identify the best suited method for a given dataset such that unbiased and valid results are yielded.

**Comment 4: There is no reason to justify Wilcoxon test as it merely stacks the order. Non parametric statistical analysis should be avoided especially since the authors explicitly compare the means.**

RESPONSE: We used the Wilcoxon test to compare the median distribution of the raw data, which is non-parametric, and confirm the model output (computed on means after standardization of the raw values)

**Comment 5: I’m not well aware of the debate between various elastic net strategies and why it should be the main focus of the publication (and take 80% of the abstract). I suggest to streamline the manuscript to make the biological results more understandable and more the center of the manuscript. Especially as far as I understood the main result is the figure 3.A. and the fact that additional innate marker yield no further information. Or did I miss something?**

RESPONSE: We agree with the reviewer and have significantly shortened the result section reporting on the MTP-EN model (lines 150).

**Reviewer #3: The authors investigated an important topic in the field of Infectious diseases. As they have pointed out the risk of tuberculosis disease is higher in individuals with recent Mycobacterium tuberculosis infection compared to individuals with more remote, established infection. Therefore, the development of tools to distinguish this patient’s population which is at higher risk for the development of TB disease could be key for disease prevention strategies.
Based on the investigators results, the determination of the level of HLA-DR on ESAT6/CFP10-specific Th1 cytokine-expressing CD4 cells has the potential to serve as a strong biomarker for recent Mycobacterium tuberculosis infection. These findings are interesting, although they require validation in larger cohorts before they can be used for clinical management.
The tree-based models showed that for E6C10-specific HLA-DR expression levels 17 out of 29 recent QFT converters could be correctly classified with values greater than or equal to -0.098. In this model, the use of this biomarker could be considered highly specific. However, the specificity for the other nodes appeared to be lower. As the decision rules identified by the tree resulted in 12% of the observations being misclassified, the strategy proposed in this manuscript could result in false classification of recent QFT converters. Nonetheless, if a significant amount of recent QFT converters can be identified (with an average AUC for the final RF model of 0.84), this strategy has the potential to be used clinically in recently exposed patients. However, if the results of this biomarker strategy suggest that a patient is not a recent QFT converter, these results should be interpreted with caution due to an average misclassification error of 0.12.
As the authors have shown in the discussion, their group and other, have shown that HLA-DR expression on Mycobacterium tuberculosis-specific T cells has been shown to have good potential as a biomarker to distinguish individuals with LTBI from those with active TB disease, and to monitor antibiotic treatment response. These results recapitulate the fact that HLA-DR expression should be studied in broader clinical samples to determine its clinical sensitivity and specificity for the detection of populations at higher risk of developing TB disease. The relevance of this biomarker also has biological sense, as HLA-DR is a cell surface receptor reflecting T cell activation.
I agree with the authors that a diagnostic test made up of a single biomarker would be simpler and more cost effective, and therefore based on the data presented in this manuscript the use of HLA-DR expression alone for the prediction of QFT recent conversion would be preferred in clinical labs. The use of complex antibody panels may be complicated and not feasible in different settings.

Comment 1: This is a paper of Tuberculosis diagnostics. However, the demographics and clinical characteristics of the subjects are not clearly described in the paper. The authors must describe extensively in a paragraph the population, and if possible, describe the clinical characteristics in a table. The manuscript would not be acceptable for publication if this point is not addressed.**

RESPONSE: We apologise for this omission. We cited another paper we recently published on the same cohort which contained detailed information. We have now added detailed information in this manuscript, lines 112 and 396, as well as in Supplementary Table 2 showing key demographic information.

**Comment 2: Would it be possible to assess if a combination of Innate and adaptive effector responses measured in stimulated PBMCs using flow cytometry, and clinical variables may help improving the discriminatory value of the tree-based models. I don’t necessarily expect this to be the case, and larger cohorts may be needed to answer this question, but some individual-specific variables such as time since tuberculosis exposure, nutritional status, and receiving preventive therapy may influence the QFT conversion patterns. The authors should at least describe these variables in this patient population. It would be important to describe if these variables are different in the patients that are not correctly classified.**

RESPONSE: This is an important point. As we have now explained more explicitly (lines 115): “Participants were healthy for the duration of the study, and the groups were balanced for age, sex, ethnicity, school of recruitment (indicative of socio-economic status) and TB exposure, all factors that have been associated with a higher risk of *M.tb* infection in the larger cohort from which these participants were selected (S2 Table and [11]).” None of the participant received preventive therapy, according to South African guidelines, and besides BMI, which was not different, no additional nutritional information was collected.

**Comment 3: Why did the authors limit the study population to adolescents 12-to-18 year of age?**

RESPONSE: In our setting acquisition of Mtb infection (i.e. QFT conversion) and incident TB sharply rise during adolescence, with ~60% 18-years old being QFT+ [11] and prevalence of QFT+ reaching ~80% in early adulthood. It would be therefore very difficult (i.e. a massive sample size would be required) to detect QFT conversion in adults, since most individuals are already QFT+. Additionally, adolescents represent a critical group for novel interventions, such as new vaccines or preventive treatment, before the sharp rise in TB disease occurs.

**Comment 4: How did the authors select the intervals for QFT testing during the 2-years of follow-up (termed months 0, 6, 12 and 18) to determine Mycobacterium tuberculosis infection? What was the rationale to select those intervals? Was it randomly selected, or there was some rationale behind? Also, what was the rationale to only follow up patients for 2 years? There is recent literature suggesting that the risk of development of TB in pediatric populations (including adolescents) is highest within 90 days of enrolment (and presumably of exposure) (2·9% [95% CI 1·7–4·9]) (Martinez, Lancet 2020). Therefore, the authors may have missed an important timeframe for biomarker discovery as testing within the first 90 days was not performed in this study. This is a very important limitation and has to be discussed in the paper. If the authors design future studies on this matter they should take this point into account.**

RESPONSE: The intervals for QFT testing were selected based on feasibility. This study was nested in a larger cohort (n=6363) which was followed-up for 2 years to determine prevalence and incidence of TB disease and Mtb infection. Starting in 2005, this was possibly the first large longitudinal study conducted in high TB endemic settings to evaluate the diagnostic and prognostic performance of QFT, and generate epidemiological data which have been used to power vaccine efficacy trials in adolescents. The participants included in this paper were followed-up longitudinally and did not develop TB disease over 2 years. This was verified at each visit, unscheduled TB investigation visits if the participants reported symptoms, and through passive surveillance. Since TB disease lasts several months, any new disease diagnosis that was made by others between study visits would have been recorded at the next visit. The study reported in this paper did not aim nor was designed to define biomarkers for incident TB, therefore we respectfully disagree that this is a limitation.

**Comment 5: Can the authors describe briefly their protocol for cryopreservation of PBMCs? How long (average) were the cells stored? Was this protocol followed for all the patients? Are there any differences in storage times within the patients? This should be included in the supplemental material.**

RESPONSE: We added the following text in the supplemental material as requested (lines 13). “PBMCs were isolated using CPT tubes (BD Biosciences), washed twice and cryopreserved in 50%, RPMI, 40% fetal bovine serum and 10% DMSO in liquid nitrogen. Samples were collected between 2006 and 2008, and thawed between 2017-2019, therefore have been cryopreserved for 9-13 years. Average post-rest viability of thawed cells was excellent (97%). “

**Comment 6: Strengths:
-The authors have defined a population longitudinally based on the QFT conversion patterns. The authors defined recent QFT converters, as individuals with two consecutive QFT negative results followed by consecutive two QFT positive results, which makes this a very well-defined population. Moreover, the need for two consecutive QFT negative and then positive results, decreases the risks of false positive and false negative results. This is a unique population which is ideal for this study.
-The authors have developed their own filtering method to robustly identify biologically meaningful cell subsets as COMPASS and MIMOSA may not be appropriate to pre-filter innate variables.**

RESPONSE: We thank the reviewer for highlighting these strengths.

**Comment 7: Limitations:
The authors only studied Mycobacterium tuberculosis infected adolescents. As it has been extensively described in the literature, the natural history of disease in tuberculosis is impacted by the age of the individuals, and well-defined phenotypes occur at different ages. Adolescents usually present with adult-type disease (Martinez, Lancet 2020; Perez-Velez NEJM 2012). Therefore, the findings reported in this study may not be applicable to other patient populations of different ages. This should be described extensively in the discussion. Presumably the prediction value of the biomarkers described in this study may not work for younger patients, or even for adult populations.**

RESPONSE: We agree with the reviewer that we only focused on a specific age group, which, as discussed in response to comment 3, is a critical target group for novel interventions in our high transmission setting. We are currently conducting a prospective cohort study to assess the performance of this biomarker in children under 5 years of age and are collaborating with other groups who are investigating adult house-hold contacts in middle- and low-transmission settings. We added the following sentence to the discussion (lines 371):

“However, due to the small sample size in this study, further validation in a large independent cohort is required, **as well as assessment of the biomarker performance in different populations, including other age groups, individuals living with HIV and low transmission settings**.”

**Have all data underlying the figures and results presented in the manuscript been provided?**

Large-scale datasets should be made available via a public repository as described in the *PLOS Computational Biology* [data availability policy](https://eur01.safelinks.protection.outlook.com/?url=https%3A%2F%2Fprotect-za.mimecast.com%2Fs%2FCyPGCBgX56fXEjkYczLnJq&data=04%7C01%7CLLYTES001%40myuct.ac.za%7Cfa1d384fb8c441cf9d4608d90680a7cb%7C92454335564e4ccfb0b024445b8c03f7%7C0%7C0%7C637547971909882514%7CUnknown%7CTWFpbGZsb3d8eyJWIjoiMC4wLjAwMDAiLCJQIjoiV2luMzIiLCJBTiI6Ik1haWwiLCJXVCI6Mn0%3D%7C1000&sdata=UNqdujW1R1HHc8nyOW75%2FUuZliH8o7RA3zJL1Ja2yjU%3D&reserved=0), and numerical data that underlies graphs or summary statistics should be provided in spreadsheet form as supporting information.

**Have the authors made all data and (if applicable) computational code underlying the findings in their manuscript fully available?**
The [PLOS Data policy](https://eur01.safelinks.protection.outlook.com/?url=https%3A%2F%2Fprotect-za.mimecast.com%2Fs%2F5GVMCGZXj8tNjkPvcQ61uZ&data=04%7C01%7CLLYTES001%40myuct.ac.za%7Cfa1d384fb8c441cf9d4608d90680a7cb%7C92454335564e4ccfb0b024445b8c03f7%7C0%7C0%7C637547971909892504%7CUnknown%7CTWFpbGZsb3d8eyJWIjoiMC4wLjAwMDAiLCJQIjoiV2luMzIiLCJBTiI6Ik1haWwiLCJXVCI6Mn0%3D%7C1000&sdata=QRPWPoFYi1YNP9v0ahuQnRAGp7XKOEBCfZ7XTnzdEFE%3D&reserved=0) requires authors to make all data and code underlying the findings described in their manuscript fully available without restriction, with rare exception (please refer to the Data Availability Statement in the manuscript PDF file). The data and code should be provided as part of the manuscript or its supporting information, or deposited to a public repository. For example, in addition to summary statistics, the data points behind means, medians and variance measures should be available. If there are restrictions on publicly sharing data or code —e.g. participant privacy or use of data from a third party—those must be specified.

Reviewer #2: **No:** i could not find but maybe there is

RESPONSE: The data is available here: <https://doi.org/10.25375/uct.13693699.v2>

The code can be found here: <https://doi.org/10.25375/uct.14573088.v1>

Figure Files:

While revising your submission, please upload your figure files to the Preflight Analysis and Conversion Engine (PACE) digital diagnostic tool, [https://pacev2.apexcovantage.com](https://eur01.safelinks.protection.outlook.com/?url=https%3A%2F%2Fprotect-za.mimecast.com%2Fs%2FT1pRCJZKmQtW0Z2NUvYsbS&data=04%7C01%7CLLYTES001%40myuct.ac.za%7Cfa1d384fb8c441cf9d4608d90680a7cb%7C92454335564e4ccfb0b024445b8c03f7%7C0%7C0%7C637547971909902498%7CUnknown%7CTWFpbGZsb3d8eyJWIjoiMC4wLjAwMDAiLCJQIjoiV2luMzIiLCJBTiI6Ik1haWwiLCJXVCI6Mn0%3D%7C1000&sdata=caLzG%2FiP2KZMgDSo%2FeGZ9NBVF0VxdMCW65TKTd2%2F8d0%3D&reserved=0). PACE helps ensure that figures meet PLOS requirements. To use PACE, you must first register as a user. Then, login and navigate to the UPLOAD tab, where you will find detailed instructions on how to use the tool. If you encounter any issues or have any questions when using PACE, please email us at [figures@plos.org](mailto:figures@plos.org).

Data Requirements:

Please note that, as a condition of publication, PLOS' data policy requires that you make available all data used to draw the conclusions outlined in your manuscript. Data must be deposited in an appropriate repository, included within the body of the manuscript, or uploaded as supporting information. This includes all numerical values that were used to generate graphs, histograms etc.. For an example in PLOS Biology see here: [http://www.plosbiology.org/article/info:doi/10.1371/journal.pbio.1001908#s5](https://eur01.safelinks.protection.outlook.com/?url=https%3A%2F%2Fprotect-za.mimecast.com%2Fs%2FuVopCKO7nQIy3OYViGXveq&data=04%7C01%7CLLYTES001%40myuct.ac.za%7Cfa1d384fb8c441cf9d4608d90680a7cb%7C92454335564e4ccfb0b024445b8c03f7%7C0%7C0%7C637547971909902498%7CUnknown%7CTWFpbGZsb3d8eyJWIjoiMC4wLjAwMDAiLCJQIjoiV2luMzIiLCJBTiI6Ik1haWwiLCJXVCI6Mn0%3D%7C1000&sdata=mc%2BMxno1%2BZr40Qh4ysxVfDGGUi9vnCP%2B%2FNzAgSjN6Fc%3D&reserved=0).

Reproducibility:

To enhance the reproducibility of your results, we recommend that you deposit your laboratory protocols in protocols.io, where a protocol can be assigned its own identifier (DOI) such that it can be cited independently in the future. Additionally, PLOS ONE offers an option to publish peer-reviewed clinical study protocols. Read more information on sharing protocols at [https://plos.org/protocols?utm_medium=editorial-email&utm_source=authorletters&utm_campaign=protocols](https://eur01.safelinks.protection.outlook.com/?url=https%3A%2F%2Fprotect-za.mimecast.com%2Fs%2Fs9pXCLg1oJfDEKg6UrmlMH&data=04%7C01%7CLLYTES001%40myuct.ac.za%7Cfa1d384fb8c441cf9d4608d90680a7cb%7C92454335564e4ccfb0b024445b8c03f7%7C0%7C0%7C637547971909912493%7CUnknown%7CTWFpbGZsb3d8eyJWIjoiMC4wLjAwMDAiLCJQIjoiV2luMzIiLCJBTiI6Ik1haWwiLCJXVCI6Mn0%3D%7C1000&sdata=6Z%2FMTbw1cXzDuWntUAx8nRnr2ps3Rpjn0roHYnZpKiU%3D&reserved=0)
